# Effect of Hydroxytyrosol on OTULIN Levels in Testıcular Tıssue in Experımental Dıabetes Model Induced wıth Streptozotocin

**DOI:** 10.1007/s43032-026-02085-9

**Published:** 2026-03-19

**Authors:** Tuba Yalçın, Sercan Kaya

**Affiliations:** Vocational Higher School of Healthcare Studies, Batman University, Main Campus, Health Services Vocational School, Room 212, Kültür Neighborhood, Batman, Turkey

**Keywords:** Diabetes mellitus, Hydroxytyrosol, OTULIN, Testis

## Abstract

Diabetes mellitus (DM), a metabolic disease, is an important health problem with many complications, including male reproductive disorders. Hydroxytyrosol (HT), an active polyphenolic, is one of the compounds of olives, which has many proven health benefits. The purpose of this study was to ascertain the possible therapeutic benefits of HT on testicular tissue in model of DM caused by streptozotocin (STZ). In addition, the aim was to investigate the effects of HT on proinflammatory cytokines and OTULIN levels in testicular tissue in an experimental DM model. A total of 32 male rats were used in the 6-week study and the rats were divided equally into 4 groups. The control group was untreated, while rats in the DM group received a single dose of STZ to induce diabetes. After inducing experimental diabetes in the DM + HT group, 5 mg/kg/day Hydroxytyrosol was administered. The HT group received 5 mg/kg/day Hydroxytyrosol. After all treatments were completed, the experiment was terminated and testicular tissues were taken for analyses. Experimental DM model caused oxidative stress, histopathological changes, increase in pro-inflammatory cytokines, decrease in OTULIN levels and increase in apoptotic germ cells in testicular tissues. In contrast, HT supplementation to rats with experimental DM was found to modulate these adverse effects to a great extent. In conclusion, HT may have anti-inflammatory, anti-oxidant and anti-diabetic effects against DM and related damage in testicular tissues by regulating OTULIN levels.

## Introduction

The hallmark of diabetes mellitus (DM), a metabolic illness, is hyperglycemia. Male reproductive impairment is one of the main complications associated with long-term uncontrolled DM, which has become a worldwide problem [1]. Approximately 90% of male patients with DM experience varying degrees of reproductive or fertility problems that significantly affect their quality of life, mental and physical health [2]. Features of DM-related testicular dysfunction include morphological changes in testicular tissue, spermatogenic dysfunction, reduced spermatogenic cell number, and sex hormone disorders [3]. Oxidative stress, inflammation, and apoptosis are the main complications that trigger organ failure in DM and have been proven to negatively affect the male reproductive system [4]. Spermatozoa are highly susceptible to oxidative damage because they are dependent on existing antioxidant protection in the reproductive system and lack ROS scavenging enzymes [5]. Both nuclear and mitochondrial DNA fragmentation in sperm cells are markedly increased by oxidative damage, which is a major factor in the pathophysiology of DM. In addition, excessive ROS increase in the cell causes a negative effect on sperm concentration, quality and functions by increasing the accumulation of lipid peroxidation products and accelerating apoptotic mechanisms [6].

Natural substances that have been shown to be safe have been used extensively in recent years to treat a wide range of diseases. These substances are very interesting, particularly those with potent anti-oxidant and anti-inflammatory properties [7]. Hydroxytyrosol (HT), one of these natural compounds, is an active polyphenolic component of olives with many proven health benefits [8]. Recently, HT has received increasing attention due to its many biological properties such as anti-inflammatory, antioxidant [9], cytoprotective [10], neuroprotective [11], and anti-obesity effects [12]. In addition, studies have reported that HT treatment improves sperm parameters, antioxidant capacity, and enzyme activity in rat testicular tissue [13, 14].

The regulation of cell-intrinsic immunological responses, including those that contain the nuclear factor kappa B (NF-κB) signaling pathway, depends on Met1-linked linear polyubiquitination (M1-Ub). When tumour necrosis factor-a (TNFα) receptors are activated, a signalling complex is formed that involves the linear ubiquitination of many target proteins to help activate the NF-κB pathway. This subsequently triggers the nuclear transactivation of genes related to cell survival, proliferation, and inflammation [15, 16]. Deubiquitinases (DUB) are proteases that act on ubiquitin ligases and cleave ubiquitins from their protein substrates [17]. The only vertebrate DUB that has been identified to have selectivity for M1-Ub chains is OTULIN [18]. On the other hand, OTULIN binds to the linear ubiquitin chain assembly complex (LUBAC) and uses the catalytic OTULIN domain to eliminate IκB-bound M1-Ub chains and suppress NF-κB signalling [19]. A study emphasized that OTULIN levels are important in regulating the inflammatory activities of NF-κB and TNF-α signaling pathways [20]. In a similar vein, another study found that OTULIN may block the TNF-α-induced NF-κB pathway by eliminating the linear ubiquitin chain [21].

Considering that inflammation, oxidative stress, and apoptosis are associated with DM-induced testicular dysfunction [22], HT, which has proven anti-inflammatory and anti-oxidant effects, has a high potential to regulate DM-induced adverse effects in testicular tissue. In this study, we aimed to detect how HT affects oxidative parameters, inflammatory and apoptotic markers in testicular tissue, and its relationship with OTULIN levels in the Streptozotocin (STZ)-induced experimental DM model.

## Material and Method

### Design of Experiments

The Fırat University Animal Experiments Ethics Committee granted authorisation for the study to begin on June 23, 2022, with the number 9340. All applications in the experimental design were carried out in accordance with the ARRIVE guidelines. 32 male Sprague-Dawley rats, ages 8 to 10 weeks, were split into 4 groups at random, with 8 rats in each of the groups listed below. Animals were housed under appropriate conditions (21–24 °C temperature, ad-libitum water and feed, 12-hour cycle) at the center where they were obtained throughout the experiment. Control (*n* = 8), rats in this group were not treated during the experiment. DM (n:8), rats in this group received a single intraperitoneal dose of STZ (50 mg/kg, pH 4.5 dissolved in phosphate-citrate buffer, Sigma) to induce experimental DM. Fasting blood glucose levels were determined 72 h after STZ administration with a blood sample taken from the tail vein of fasted (12 h) rats. Rats that had fasting blood glucose levels more than 250 mg/dL were considered to have experimental DM. DM + HT (*n* = 8), after experimental DM was induced with STZ, 5 mg/kg HT (Sigma Chemical) was administered to rats in this group at the same time every day for 6 weeks using orogastric tube. HT (*n* = 8) rats in this group received 5 mg/kg/day HT throughout the experiment. Taking the studies into consideration, the experimental design’s recommended STZ and HT dosages and uses were established [23, 24]. The experiment was terminated at the end of 6 weeks by sacrificing the rats under anaesthesia (xylazine/ketamine, 10/75 mg/kg) and removing the testicular samples.

### Histopathological Examinations

The right testicular tissue was quickly preserved in Bouin’s solution for histological and immunohistochemical analyses at the conclusion of the experiment. Following fixation, testicular tissues underwent a number of histological follow-up procedures, were embedded in paraffin blocks, and then had testicular tissue sections that were 4–5 μm thick. Hematoxylin-eosin (HE) was used to stain testicular tissue sections for histopathological analysis. Histopathological examination and imaging were performed with an optical microscope (Leica-DM 2500, Germany). Histopathological evaluation of HE-stained testicular sections was performed by considering the presence of seminiferous tubule degeneration, germinal epithelial detachment, and vacuolization in seminiferous tubules. The histopathological evaluation score was calculated by evaluating ten different non-overlapping regions at x10 magnification and according to the presence of the criterion, a maximum score of 9 points was calculated with 3 graded scores (0 = none, 1 = less, 1 = less, 2 = medium, 3 = more). Additionally, the Johnsen score (JS), which evaluates the progression of spermatogenesis, the loss of mature spermatogenic cells following testicular injury, and germinal epithelial degeneration, was computed [25]. In testicular tissue sections, 40 different seminiferous tubules that did not overlap were randomly selected and scored in the range of 1–10. In short, seminiferous tubules with normal histological structure and complete spermatogenesis were given 10 points, while seminiferous tubules without Sertoli and spermatogenic germ cells were given 1 point and JS was calculated [26].

### Immunohistochemical Investigations

The Avidin Biotin Peroxidase Complex (ABC) method was used to measure the immunoreactivities of Caspase-3 (Casp3; Bioss, China), Cleaved Casp3 (Asp175; MedChem Express USA), and OTULIN (Boster, CA) in testicular tissue sections in accordance with the previously detailed protocol [27]. Testicular sections were counterstained with Mayer’s haematoxylin and examined with an optical microscope (DM 2500-Leica, Germany). Casp3 Cleaved Casp3, and OTULIN immunoreactivities were calculated by multiplying the prevalence of immunostaining by the intensity of immunostaining [28].

$$\boldsymbol I\boldsymbol m\boldsymbol m\boldsymbol u\boldsymbol n\boldsymbol o\boldsymbol r\boldsymbol e\boldsymbol a\boldsymbol c\boldsymbol t\boldsymbol i\boldsymbol v\boldsymbol i\boldsymbol t\boldsymbol y\;=\;Immunoreactivity\;prevalence\;\boldsymbol X\;Immunoreactivity\;intensity$$

Immunohistochemical evaluations were calculated using the formula described above in 10 different nonoverlapping areas at x20 magnification to determine Casp3, Cleaved Casp3, and OTULIN immunoreactivities [29].

### TUNEL Assay

DNA fragmentation can be found using a technique called TUNEL. This method allows for the identification of double-stranded DNA breaks during apoptosis by labelling the 3’ hydroxyl terminus. Apoptotic cells in testicular tissue were detected using the ApopTagPlus Peroxidase In Situ Apoptosis Detection Kit (Chemicon, USA, Cat: S7101). Cells with blue nuclei were regarded as healthy in the TUNEL assay evaluation, while cells with brown or black nuclei were regarded as TUNEL-positive apoptotic cells. The apoptotic index (%) was calculated by counting 250 cells in 25 randomly selected areas [30, 31].

### Biochemical Analyses

Left testicular tissues taken at the end of the experiment and stored at -80 °C for analysis were homogenized by centrifugation (+ 4 °C, 5000 rpm, phosphate buffer solution 10%, 15 min). The supernatants obtained from homogenised testicular tissues were studied by ELISA (Enzyme-Linked Immunosorbent Assay) method. Catalase (CAT), Malondialdehyde (MDA), Superoxide Dismutase (SOD), Glutathione (GSH), NF-κB, OTULIN, and TNFα levels were measured using the ELISA technique. The firms supplied the CAT, MDA, SOD (FineTest, China), OTULIN (Sunred, China), GSH, NF-κB, and TNFa (BT Lab., China) ELISA kits, which are capable of quantitative measurements in the tissue samples specific to rats utilised in the study. ELISA tests were performed in accordance with the manufacturer’s instructions.

### Statistical Analyses

The SPSS 22.0 program was used to perform statistical analysis of the data. To ascertain if the data followed a normal distribution, the Shapiro-Wilk test was employed. Data that did not show a normal distribution were analysed using the Kruskal-Wallis and Mann-Whitney U tests. Data with a normal distribution were analysed using one-way ANOVA post-hoc Tukey tests. A p-value of less than 0.05 was established as the statistical significance criterion. The graphs were made using the Graph-Pad Prism 8 program.

## Results

### Impact of DM and/or HT on Testicular Tissue Oxidative Stress Metrics

**Fig. 1:**
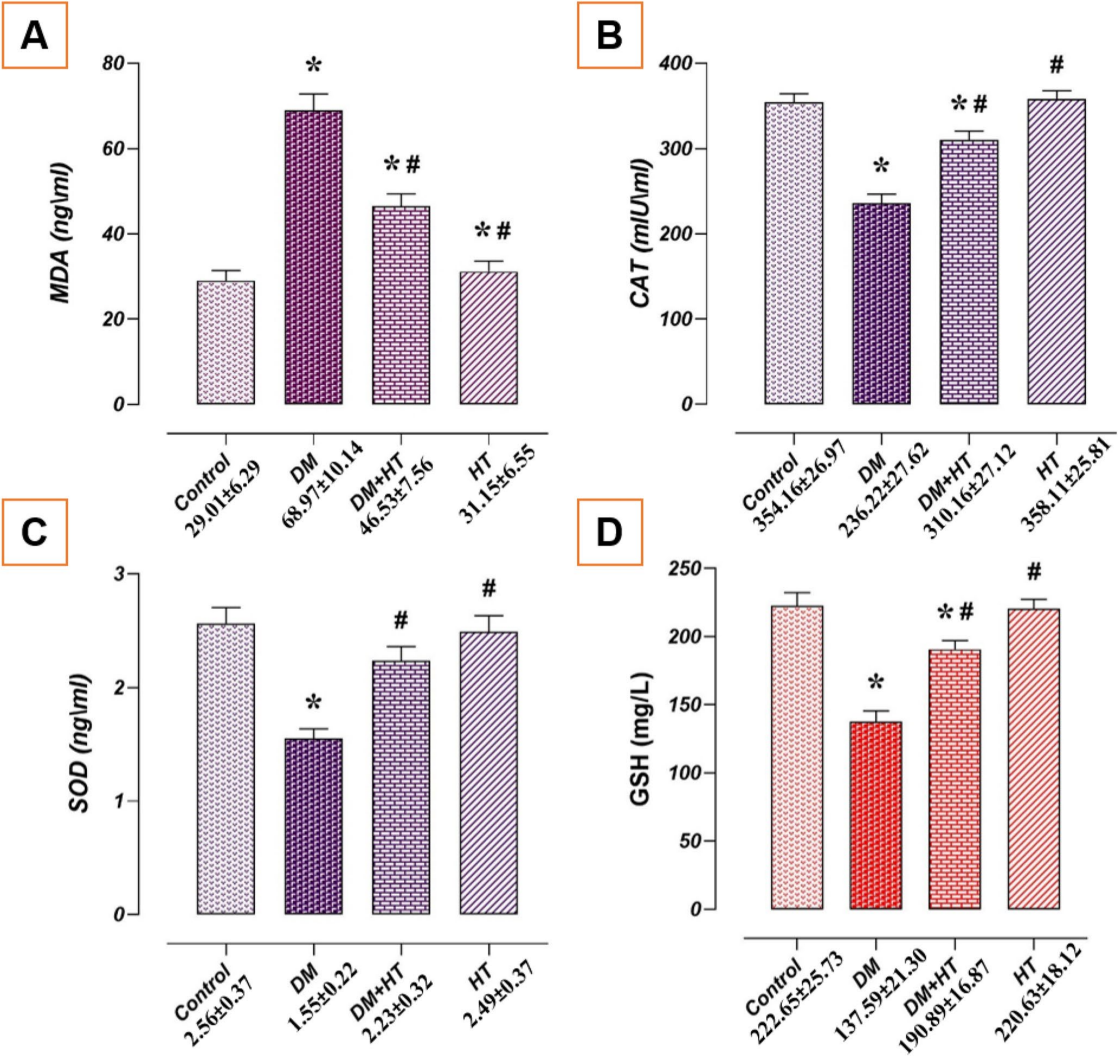
Effect of DM and/or HT on oxidative stress parameters in testicular tissue. The testicular tissues of the DM and control groups had comparable oxidant/antioxidant characteristics. Comparing the DM group to the control group, MDA levels rose while CAT, GSH, and SOD levels fell. Compared to the DM group, the DM + HT group had higher levels of CAT, GSH, and SOD and lower levels of MDA. **A** MDA level, **B** CAT level, **C** SOD level, **D** GSH level. *: compared with the control group (*p* < 0.05). #: compared with the DM group (*p* < 0.05). Data are presented as mean±standard deviation (ANOVA). *DM* Diabetes mellitus, *HT* Hydroxytyrosol, *CAT* Catalase, *MDA* Malondialdehyde, *GSH* Gulutathione, *SOD* Superoxide dismutase

Oxidant-antioxidant indicators showed differences between the research groups (*p* < 0.05). The control and HT groups’ oxidative stress metrics in testicular tissues were similar (*p* > 0.05). Comparing the DM group to the control group revealed that whereas CAT, SOD, and GSH levels declined (*p* < 0.05), MDA levels increased. Comparing the DM + HT group to the DM group, MDA levels declined while GSH, SOD, and CAT levels increased (*p* < 0.05) (Fig. 1).

### Effect of DM and/or HT on Testicular Tissue Histopathology

**Fig. 2:**
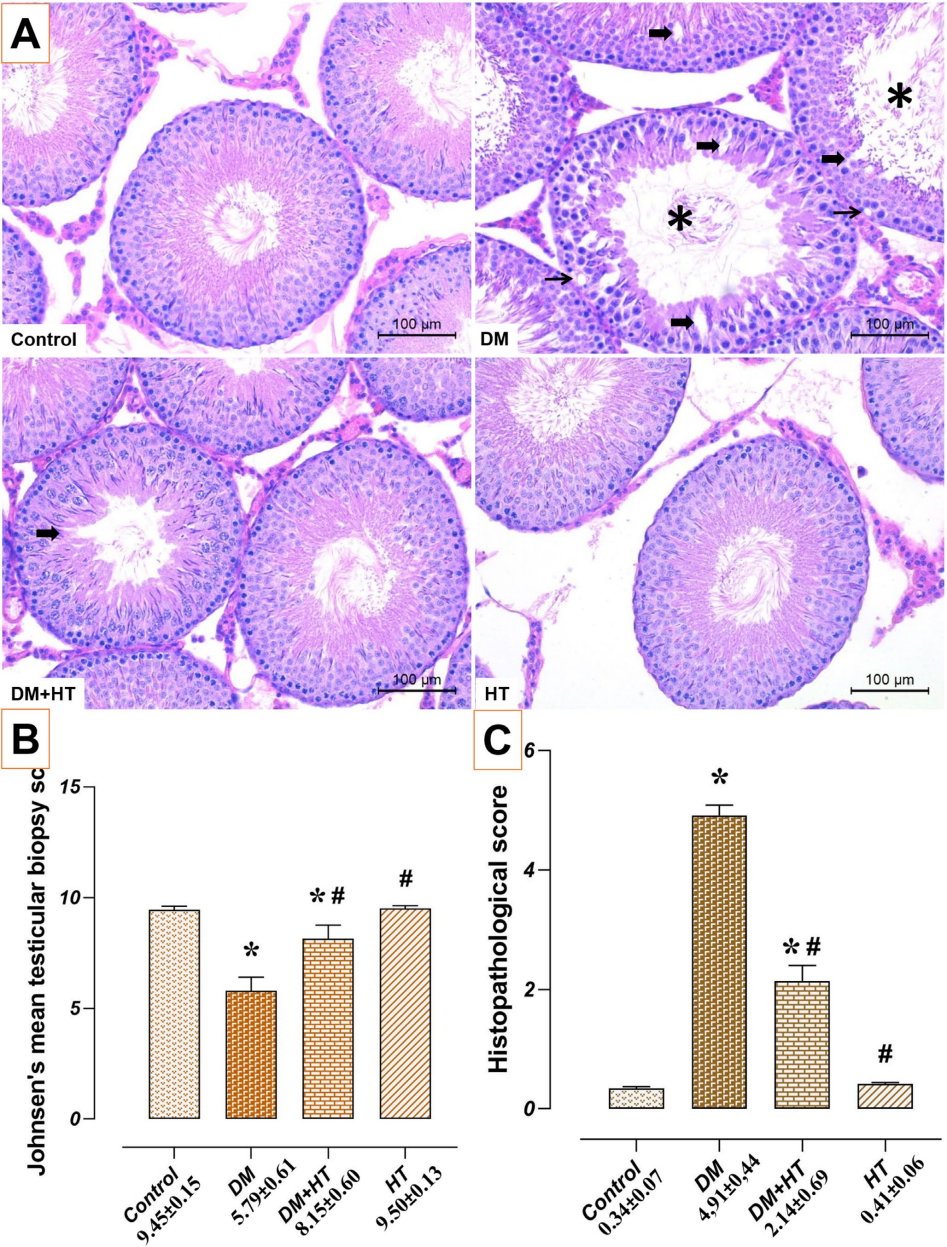
Effect of DM and/or HT on testicular tissue histopathology. Normal histological structure, similar Johnsen’s score and histopathological score were observed in testicular tissues of control and HT groups. In comparison to the control group, Johnsen’s score dropped and the histopathological score rose with diffuse histological alterations in the DM group. In comparison to the DM group, the Johnsen score rose, the histopathological score fell, and the histopathological alterations considerably decreased in the DM + HT group. *: compared to control group (*p* < 0.05). #: compared to DM group (*p* < 0.05). Data are presented as mean±standard deviation (ANOVA). **A** Haematoxylin eosin, scale bar 100 μm, **B** Johnsen score graph, **C** Histopathological score graph. Asterisk: Seminiferous tubule degeneration, Thick arrow: Germinal epithelial separation, Thin arrow: Vacuolisation, *DM* Diabetes mellitus, *HT* Hydroxytyrosol

Both the control and HT groups’ testicular tissues displayed the normal histological structure. Testicular tissues of the DM group showed severe histopathological changes, especially seminiferous tubule degeneration, compared to the control group (*p* < 0.05). However, histopathological changes were significantly reduced in the DM + HT group compared to the DM group (*p* < 0.05). In line with these findings, the DM group’s histological evaluation score increased and JS decreased when contrasted to the control group (p 0.05). When comparison to the DM group, the DM + HT group’s histological evaluation score decreased and JS increased (*p* < 0.05) (Fig. 2).

### Impact of DM and/or HT on Testicular Tissue Levels of TNFα and NF-κB

**Fig. 3:**
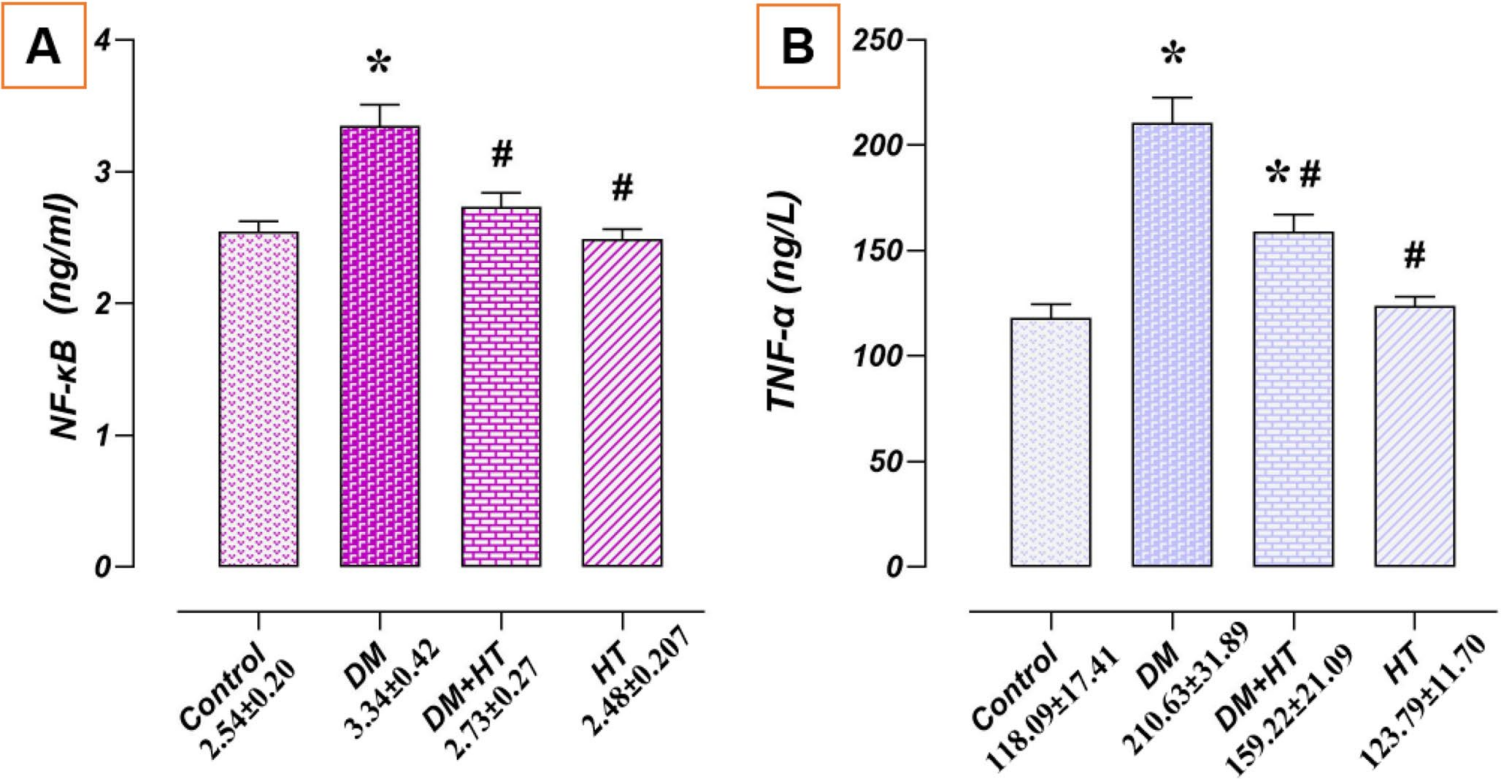
Effect of DM and/or HT on NF-κB and TNFα levels in testicular tissue. The levels of TNFα and NF-κB in the testicular tissues of the HT and control groups were comparable. The DM group had higher levels of TNFα and NF-κB than the control group. NF-κB and TNFα levels were lower in the DM + HT group than in the DM group. **A** NF-κB levels, **B** TNFα levels. *; compared to control group (*p* < 0.05). #; compared to DM group (*p* < 0.05). Data are presented as mean±standard deviation (ANOVA). *DM* Diabetes mellitus, *HT* Hydroxytyrosol, *NF-κB* Nuclear factor kappa B, *TNFα* Tumour necrosis factor a

The research groups’ levels of pro-inflammatory cytokines differed significantly (*p* < 0.05). The HT and control groups’ testicular tissues had comparable amounts of TNFα and NF-κB (*p* > 0.05). The DM group had higher levels of TNFα and NF-κB than the control group (*p* < 0.05). NF-κB and TNFα levels decreased (*p* < 0.05) in the DM + HT group compared to the DM group (Fig. 3).

### Impact of HT and/or DM on Testicular Tissue OTULIN Levels

**Fig. 4:**
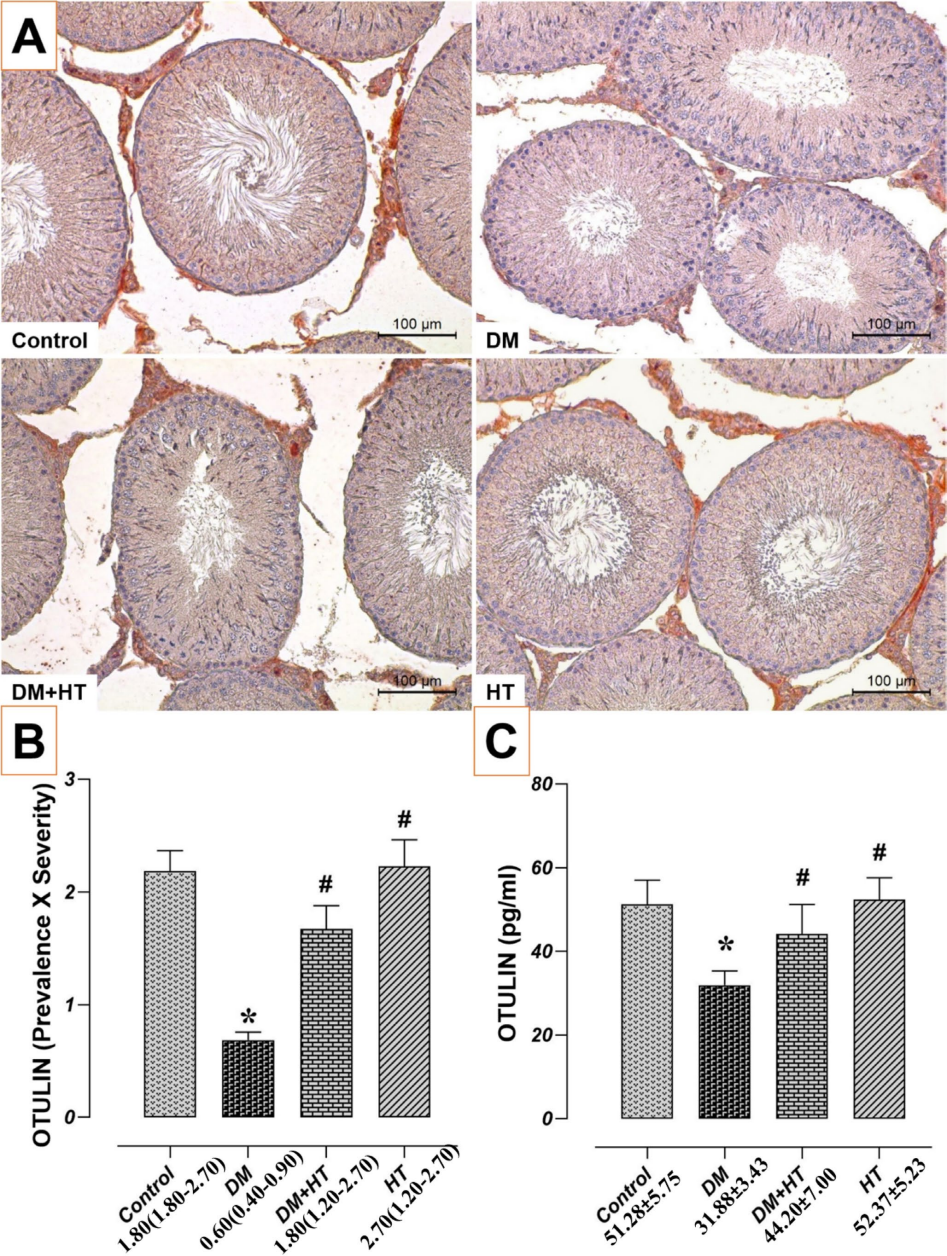
Effect of DM and/or HT on OTULIN levels in testicular tissue. Testicular tissues from the HT and control groups had comparable amounts of OTULIN. The DM group’s OTULIN levels were lower than those of the control group. The DM + HT group had higher levels of OTULIN than the DM group. *: compared to control group, #: compared to DM group (*p* < 0.05). **A** OTULIN immunohistochemical staining microphotographs (scale bar; 100 μm), **B** OTULIN immunoreactivity graph, data are presented as median (min - max) (Kruskal Wallis). **C** OTULIN testis ELISA levels, data are presented as mean±standard deviation (ANOVA). *HT* Hydroxytyrosol, *DM* Diabetes mellitus

OTULIN immunoreactivities and ELISA levels were comparable in the HT and control groups (*p* > 0.05). OTULIN levels were lower in the DM group than in the control group (*p* < 0.05). On the other hand, OTULIN levels rose (*p* < 0.05) in the DM + HT group relative to the DM group (Fig. 4).

### HT and/or DM’s Impact on Testicular Tissue Apoptotic Indicators

**Fig. 5:**
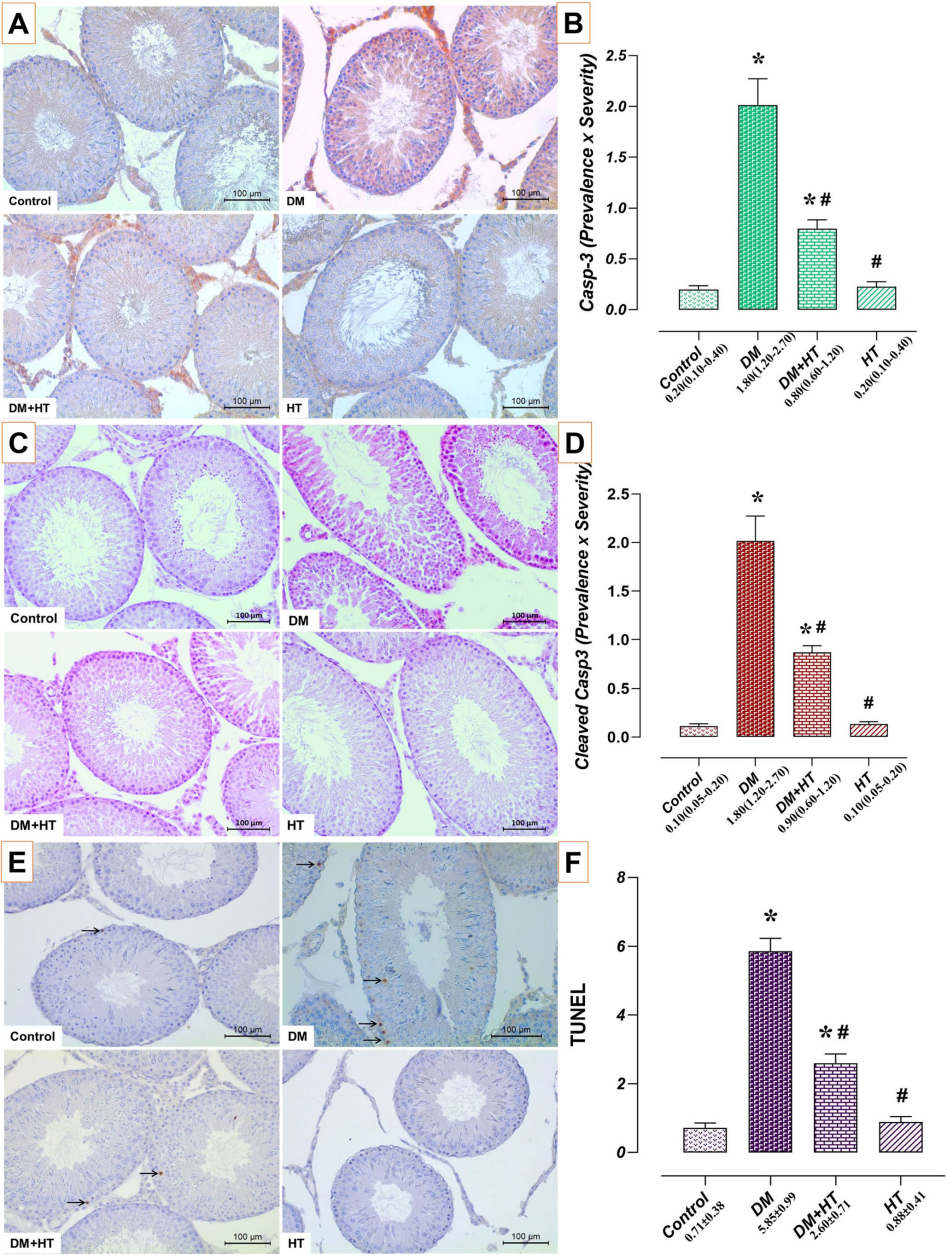
Impact of HT and/or DM on testicular tissue apoptotic indicators. Testicular tissues from the control and HT groups showed comparable amounts of TUNEL-positive apoptotic cells, Casp3, and Cleaved Casp3 immunoreactivities. The DM group had higher numbers of TUNEL-positive apoptotic cells, Casp3, and Cleaved Casp3 immunoreactivities than the control group. TUNEL-positive apoptotic cell levels, Casp3, and Cleaved Casp3 immunoreactivities were lower in the DM + HT group than in the DM group. *: compared to control group, #: compared to DM group (*p* < 0.05). **A** Casp3 immunohistochemical staining microphotographs (scale bar; 100 μm), **B** Casp3 immunoreactivity graph, data are presented as median (min-max) (Kruskal Wallis). **C** Cleaved Casp3 immunohistochemical staining microphotographs (scale bar; 100 μm), **D** Cleaved Casp3 immunoreactivity graph, data are presented as median (min-max) (Kruskal Wallis). **E** TUNEL staining microphotographs (scale bar; 100 μm), **F** TUNEL positive - Apoptotic Index, data are presented as mean±standard deviation (ANOVA). Arrow: TUNEL positive apoptotic cells, *DM* Diabetes mellitus, *HT* Hydroxytyrosol

Proapoptotic Casp3, Cleaved Casp3 immunoreactivity, and the apoptotic index (% TUNEL positive apoptotic cell ratio) were comparable in the testicular tissues of the HT and control groups (*p* > 0.05). The DM group had greater levels of apoptotic index, Casp3, and Cleaved Casp3 immunoreactivity than the control group (*p* < 0.05). The DM + HT group had lower levels of apoptotic index, Casp3, and Cleaved Casp3 immunoreactivity than the DM group (*p* < 0.05) (Fig. 5).

## Discussion

In an experimental DM model produced by STZ (50 mg/kg), we have recently demonstrated that fasting blood glucose levels raised and insulin levels declined. In the same study, we reported that these adverse effects of experimental DM and histopathological changes in pancreatic tissue were significantly corrected by HT supplementation (5 mg/kg) [24]. However, DM causes oxidative stress by suppressing antioxidant mechanisms and increasing ROS in many tissue models [32]. Oxidative stress is effective in DM progression by experimental studies in rats and in patients with DM. The majority of research has demonstrated that the primary cause of male diabetics’ reproductive failure is testicular oxidative stress brought on by the disease [33, 34]. The use of antioxidant substances is recommended in the treatment of DM [35]. Antioxidants like vitamin E, vitamin C, and α-lipoic acid have been demonstrated in recent research to lessen the harm that oxidative stress causes [36]. Thus, the purpose of this study was to assess HT’s potential therapeutic impact in a rat model of DM.

In the present study, significant decreases in antioxidant enzyme activities and an increase in lipid peroxidation product (MDA) and decreases in CAT, GSH, and SOD related to antioxidant capacity were found in the testes of rats with DM. After that, the free radical superoxide is transformed into even more reactive molecules that have the potential to harm cells. The typical reaction to this oxidative damage is the spread of SOD, which breaks down the oxygen free radical into H_2_O_2_, which CAT and GPx subsequently transform into H_2_O and O_2_. This explains why the DM group in this study had lower antioxidant enzyme activity. It is commonly known that inflammation and oxidative stress are related. Reactive species released by immune-activated cells at the site of inflammation trigger and intensify intracellular signalling pathways, which in turn promote the synthesis of genes that promote inflammation. DM is regarded as an inflammatory illness in addition to alterations in metabolic state. Hyperglycaemia induces the production of growth factors, ROS, adhesion molecules, and cytokines that activate transcription NF-κβ, which controls gene expression during inflammation. This results in several organs malfunctioning [37]. As shown in a study, high levels of IL-1β, TNF-α, and IL-6 were associated with functional impairment in the testes and accessory glands [38].

The activation of transcription factors like NF-κB, which is crucial for inflammation and iNOS activation, is one of the targets of chronic oxidative stress, as seen in DM [39]. Activation of proinflammatory mediators has been reported in the testes of diabetic rats. In particular, upregulation of TNF-α, NF-κB, iNOS, and IL-6 was observed in the testes of diabetic rats [33, 40]. In the present study, NF-κB and TNF-α, which are inflammation markers, increased in testicular tissue homogenate. When activated by ROS, NF-kB upregulates TNF-α expression through activation of Casp8, which directs the extrinsic apoptotic pathway, and this triggers inflammatory responses and apoptosis [41]. Supporting this result, we noticed that the DM group’s TUNEL and Casp3 immunoreactivity were higher than the control group’s, indicating an increase in apoptotic signals.

OTULIN decreases the amount of M1 ubiquitin that LUBAC produces in the cytosol [42], but it also increases LUBAC activity by stopping its autoubiquitination [43]. Anti-TNF neutralising antibodies can be used to treat OTULIN-associated auto-inflammatory syndrome (ORAS), a potentially lethal autoinflammatory disease that occurs in patients with homozygous loss-of-function mutations in OTULIN [42, 44]. Studies on tissue-specific targeting have highlighted OTULIN’s significance in regulating NF-κB and cell death responses [45–47]. Our findings showed that OTULIN levels decreased in the DM group compared to the control group.

The literature has highlighted the presence of apoptosis, aberrant spermatozoa, and reduced testosterone levels in testicular germ cells in diabetic animal models [48]. In a different study, the majority of the spermatogonia displayed cytoplasmic vacuolization, and the testicular tissue of diabetic mice displayed giant cell development, intertubular haemorrhage, irregular seminiferous tubules, and maturation arrest. Spermatogenic cells in the tubule lumen of diabetic rats similarly displayed exfoliation and degeneration [49]. In contrast to the control group, seminiferous tubules in testicular histopathology from DM showed signs of degeneration and vacuolization.

HT is a powerful antioxidant. This feature helps to prevent cellular damage by fighting free radicals. It also has the potential to reduce inflammation [50]. HT administration to DM rats decreased histopathologic changes and increased OTULIN immunoreactivity compared to DM group. Moreover, in parallel with this increase in OTULIN immunoreactivities, HT administration to DM rats also caused an increase in OTULIN tissue homogenate levels compared to the DM group. CAT activity was statistically higher in the HT group compared to the DM group. Furthermore, as compared to the DM group, the HT group considerably reduced MDA levels and raised non-enzymatic (GSH) and enzymatic (CAT, SOD) antioxidant levels. Conversely, HT supplementation dramatically decreased inflammation. Moreover, HT supplementation also regulated OTULIN levels by significantly reducing DNA damage levels in the testes of rats with DM. Rats with DM experienced a significant decrease in these adverse effects when given HT supplements. In conclusion, supplementing with HT may mitigate the effects of DM on the reproductive system of males in rats.

## Data Availability

Data will be provided upon reasonable request.
